# Co-infections and co-morbidities among injecting drug users versus sexually infected patients in Bucharest

**DOI:** 10.7448/IAS.17.4.19665

**Published:** 2014-11-02

**Authors:** Eliza Manea, Raluca Jipa, Iulia Niculescu, Serban Benea, Otilia Benea, Victoria Arama, Adriana Hristea

**Affiliations:** 1 INBI Prof Dr Matei Bals, Bucharest, Romania; 2 INBI Prof Dr Matei Bals, Umf Carol Davila Bucharest, Bucharest, Romania

## Abstract

**Introduction:**

After the 2008 introduction of new psychoactive substances (NPS) in Romania, the number of newly diagnosed HIV infections showed significant increase among injecting drug users (IDUs). Our objective was to analyze the differences between co-infections related to the HIV infection, based on the way of transmission (IDUs versus sexually infected).

**Materials and Methods:**

A retrospective transversal study was carried out, analyzing 245 adult HIV-positive patients, diagnosed between January 2013 and December 2013 in our hospital. We collected socio-demographic, epidemiological and laboratory data at the diagnosis and analyzed them using SPSS version 20.

**Results:**

Most patients (71%, 174/245) were men and the median age was 32 years (IQR: 26–38). 91 patients (37%) were former/active IDUs (most of them injecting both opioids and NPS), while 154 patients (63%) were sexually infected, with 84% being heterosexuals and 16% men having sex with men (MSM). The median CD4 count, at the moment of diagnosis, was 294 cells/mm^3^ (IQR: 119–483).

Other co-infections at diagnosis were toxoplasmosis (four patients), cryptococcosis (two patients) and cytomegalovirus reactivations (three patients) without significant association between the two groups.

**Conclusion:**

Heterosexual transmission was the most common way of HIV transmission in 2013 in contrast with EU/CEE, where MSM accounted for the majority of cases of HIV epidemics in 2012 [1]. Sexually transmitted HIV infection was associated with late presentation, stage C and syphilis. We noted a high percentage of IDU transmission, which was associated with stage A and hepatitis C infection.

**Table 1:** Patient characteristics

	IDUs (N = 91)	Sexually infected (N = 154)	p value, OR (95% CI)
Male N (%)	73 (80)	101 (66)	0.015, 0.4 (0.2–0.8)
Age (years) (median, IQR)	30 (IQR: 26–34)	34 (IQR: 26–41)	<0.001
CD4 (cells/mm^3^) (median, IQR)	350 (IQR: 154–600)	272 (IQR: 91–405)	<0.001
Late presenters (CD4 < 350) N (%)	45 (50)	102 (66)	0.01, 1.3 (1.0–1.7)
Stage A N (%)	60 (66)	60 (39)	<0.001, 3.0 (1.7–5.2)
Stage B N (%)	21 (23)	53 (34)	0.062, 0.5 (0.3–1.03)
Stage C N (%)	10 (11)	41 (27)	0.004, 2.4 (1.2–4.6)
Hepatitis C N (%)	84 (92)	8 (5)	<0.001, 255.5 (85.7–761.4)
Hepatitis B N (%)	12 (13)	19 (12)	0.822, 1.09 (0.5–2.3)
Syphilis N (%)	4 (4)	26 (17)	0.006, 3.6 (1.3–10.0)
Tuberculosis N (%)	1 (1)	13 (8)	0.064, 0.1 (0.07–1.3)
Pneumocystosis N (%)	2 (2)	7 (5)	0.709, 0.7 (0.1–4.2)

IQR = interquartile range, IDUs = injecting drug users.
